# Interleukin-1β and Interleukin-1 Receptor Antagonist Appear in Grey Matter Additionally to White Matter Lesions during Experimental Multiple Sclerosis

**DOI:** 10.1371/journal.pone.0083835

**Published:** 2013-12-23

**Authors:** Marloes Prins, Charlotta Eriksson, Anne Wierinckx, John G. J. M. Bol, Rob Binnekade, Fred J. H. Tilders, Anne-Marie Van Dam

**Affiliations:** 1 VU University Medical Center, Neuroscience Campus Amsterdam, Dept. Anatomy and Neurosciences, Amsterdam, The Netherlands; 2 UNIV UMR1052, Centre de Recherche en Cancérologie de Lyon, Lyon, France; Friedrich-Alexander University Erlangen, Germany

## Abstract

**Background:**

Multiple sclerosis (MS) has been mainly attributed to white matter (WM) pathology. However, recent evidence indicated the presence of grey matter (GM) lesions. One of the principal mediators of inflammatory processes is interleukin-1β (IL-1β), which is known to play a role in MS pathogenesis. It is unknown whether IL-1β is solely present in WM or also in GM lesions. Using an experimental MS model, we questioned whether IL-1β and the IL-1 receptor antagonist (IL-1ra) are present in GM in addition to affected WM regions.

**Methods:**

The expression of IL-1β and IL-1ra in chronic-relapsing EAE (cr-EAE) rats was examined using *in situ* hybridization, immunohistochemistry and real-time PCR. Rats were sacrificed at the peak of the first disease phase, the trough of the remission phase, and at the peak of the relapse. Histopathological characteristics of CNS lesions were studied using immunohistochemistry for PLP, CD68 and CD3 and Oil-Red O histochemistry.

**Results:**

IL-1β and IL-ra expression appears to a similar extent in affected GM and WM regions in the brain and spinal cord of cr-EAE rats, particularly in perivascular and periventricular locations. IL-1β and IL-1ra expression was dedicated to macrophages and/or activated microglial cells, at sites of starting demyelination. The time-dependent expression of IL-1β and IL-1ra revealed that within the spinal cord IL-1β and IL-1ra mRNA remained present throughout the disease, whereas in the brain their expression disappeared during the relapse.

**Conclusions:**

The appearance of IL-1β expressing cells in GM within the CNS during cr-EAE may explain the occurrence of several clinical deficits present in EAE and MS which cannot be attributed solely to the presence of IL-1β in WM. Endogenously produced IL-1ra seems not capable to counteract IL-1β-induced effects. We put forward that IL-1β may behold promise as a target to address GM, in addition to WM, related pathology in MS.

## Introduction

Multiple Sclerosis (MS) is a chronic demyelinating disease of the central nervous system (CNS) resulting in a wide range of neurological symptoms including impaired sensory and/or motor function [1]. Although the cause and exact pathogenesis of MS remains unclear, important pathological hallmarks of MS are the influx of leukocytes into the CNS leading to a local inflammatory environment [2]. Consequently, demyelination reduces conductance velocity within the axons, and subsequent axonal loss and neuronal damage can further contribute to functional impairment [3], [4]. Until a decade ago, MS pathology has been attributed to inflammation in and demyelinaton of the white matter (WM) resulting in white matter lesions (WML). However, the pathological observations in the white matter did not always explain or predict the clinical symptoms observed in the patients [5]. In more recent years, this clinico-radiological paradox has been largely solved by the accumulating evidence from histopathological [6]–[9] and, to some extent, by high resolution imaging studies, e.g. double inversion recovery (DIR) [10]–[14], showing that the CNS grey matter (GM) is also affected in MS patients. GM lesions (GML) can occur in various brain regions of MS patients, ranging from the cortex to deep gray matter structures. These GML may explain certain cognitive impairments or psychiatric problems that occur in a great number of MS patients already early in the disease [15], [16].

Based on post-mortem observations, GML formation is considered to differ from WML lesion formation. In general a relative absence of infiltrating leukocytes and of macrophage phagocytic activity has been described in post-mortem analyzed GML [17], [18]. This observation is best illustrated in leukocortical lesions encompassing WML and GML (type I lesions) where WML areas encompass higher levels of inflammatory cells than GML [17]. Still, activated microglial cells can be observed in or surrounding the GML [9], [19]. Of interest is the observation of infiltrating immune cells during ongoing disease as identified in biopsy material of MS patients [20]. This suggests that inflammatory activity is present in grey matter at an early stage of the disease.

One of the principal mediators of inflammatory processes is the cytokine interleukin-1β (IL-1β) [21]–[23]. IL-1β can be expressed by many cell types, including leukocytes and microglia [22], [24]. IL-1β signaling and thereby IL-1β actions, can be inhibited by the endogenous and competitive IL-1 receptor antagonist (IL-1ra), an anti-inflammatory cytokine that can be produced in inflamed tissues [23].

In the intact brain, IL-1β and IL-1ra are constitutively expressed at low levels [25], but the synthesis of both IL-1β and IL-1ra can be rapidly and strongly upregulated in discrete brain areas in response to e.g. systemic inflammation [26]–[29], excitotoxic- and ischemic brain damage [22], [24], [30], brain trauma and infections [31].

IL-1 signaling contributes to neuropathological processes such as gliosis and oligodendrocyte degeneration [32]. Furthermore, IL-1β is implicated in proliferation of macrophages [33], [34], up-regulation of cellular adhesion molecules [35] and leukocyte migration [36]. All of these processes are considered to be involved in the focal inflammatory responses and subsequent formation of WML in the CNS of MS patients, and can be mimicked in animal models of MS pathology *e.g.* in experimental autoimmune encephalomyelitis (EAE) [37].

The involvement of IL-1β in MS is substantiated by the presence of IL-1β [38] and IL-1ra [39] in WML in post-mortem brain material of MS patients and of acute or chronic EAE models of MS [40]–[42]. In addition, reduction of IL-1 action by administration of IL-1ra, reduces the neurological defects in animals subjected to EAE [43]–[45]. With the current knowledge that GML are present in the MS brain, it is unknown whether IL-1 contributing to MS/EAE pathology is just solely present in WML as has been focused upon thus far, or whether it is also present in GML.

Therefore, we questioned whether IL-1β and its antagonist IL-1ra are present in GM, in addition to WM regions affected during chronic-relapsing EAE (cr-EAE) in Dark Agouti (DA) rats. This experimental animal model mimics certain relevant clinical symptoms and inflammatory pathology associated with relapsing-remitting MS [46]. To this end, we studied IL-1β and IL-1ra mRNA in the CNS in the early stages of cr-EAE and related these to some histopathological hallmarks of inflammation in the affected grey and white matter.

## Materials and Methods

### Ethics statement

The animal experimental procedures were approved by the local animal ethical committee of the VU University, Amsterdam, The Netherlands, under protocol number Fal 00-06.

### Experimental animals

Adult male DA rats were obtained from Harlan Nederland (Horst, The Netherlands). Rats were housed 2 per cage under controlled light/dark conditions (light on at 7 am and off at 7 pm) with food and water available *ad libitum*. At the start of the experiments, the animals had a body weight of approximately 250 grams.

### Induction of remiting-relapsing EAE

The N-terminal sequence (amino acids 1–125) of rat myelin oligodendrocyte glycoprotein (MOG-peptide) was used for immunization as described [47]. Under isoflurane anaesthesia, rats were given an intradermal injection into the tail base of 200 µl of an emulsion containing 75 µg of MOG peptide, 100 µl incomplete Freund's adjuvant (IFA; Difco, Detroit, USA) and 50 µl 0.01 M sodium acetate, pH 3.0. Control animals received the emulsion without MOG peptide.

### Neurological deficits

Animals were weighed daily and assessed for neurological motor deficits starting at the day of immunization (day 0). Neurological deficits were scored on a scale from 0 to 4 in which 0 = no clinical signs, 0.5 = partial loss of tail tone, 1 = complete tail atony and unsteady gait, 2 = partial paralysis of hind limbs, 3 = complete hind limb paralysis and 4 = moribund state [48].

### Tissue preparation and processing

Rats immunized with MOG-peptide were sacrificed at different phases of the disease: at the peak of the first disease phase (day 13), the trough of the remission phase (day 17), and at the peak of the relapse (day 21). Control animals were sacrificed at 13 days or 21 days after immunization with vehicle.

#### For *in situ* hybridization experiments and CD68 immunohistochemistry

Groups of rats (first disease phase, day 13, n = 8; remission, day 17, n = 5; relapse, day 21, n = 10; controls: day 13, n = 3 and day 21, n = 4) were sacrificed, and immediately the brain and spinal cord (cervical, thoracic, lumbar and sacral segments) were dissected out, frozen on dry ice, and stored at −80°C. Series of coronal brain sections (12 µm) were prepared by using a cryostate at 0.9, 3.6, 4.8, 9.7 and 10.8 mm posterior to bregma [49]. In addition, series of coronal sections (12 µm) were prepared from cervical, thoracic, lumbar and sacral segments of the spinal cord. Sections were stored at −20°C until processing for *in situ* hybridization or immunohistochemistry. Consecutive sections were processed for *in situ* hybridization, and for CD68 or CD3 immunohistochemistry. Double-labeling experiments (*in situ* hybridization in combination with CD68 immunohistochemistry) were performed to determine the cellular identity of IL-1β and IL-1ra mRNA expressing cells.

Subsequent semi-quantitative analysis identified the relative number IL-1β or IL-1ra expressing cells as a percentage of CD68 positive cells within each brain region. We used a graded scale, ranging from 0–3 in which 0 represents the absence of IL-1β or IL-1ra labeled cells; 1 represents less than 33% of CD68 positive cells express IL-1β or IL-1ra; 2 represents between 33-66% of CD68 positive cells express IL-1β or IL-1ra; 3 represents over 66% of CD68 positive cells express IL-1β or IL-1ra.

#### For immunohistochemistry of IL-1β, IL-1ra, proteolipid protein (PLP), T-cell and Oil-Red O lipid staining

Groups of rats (first disease phase, day 13, n = 4; remission, day 17, n = 4; and relapse, day 21, n = 3; controls, day 21, n = 4) were anaesthesized with an overdose of sodiumpentobarbital and intracardially perfused with 0.2 M phosphate-buffered saline (PBS pH 7.4) followed by 4% paraformaldehyde in PBS. Brain and spinal cord segments were dissected out, postfixed for 2.5 h at 4°C in the same fixative and subsequently incubated overnight at 4°C in 10% sucrose in PBS. Coronal brain sections (12 µm) were cut. In addition, series of coronal sections were prepared from cervical, thoracic, lumbar and sacral spinal cord segments. Sections were stored at −20°C until processed for immunohistochemistry.

### *In situ* hybridization histochemistry for IL-1β and IL-1ra

*In situ* hybridization histochemistry was performed according to the protocol of Young [50]. Briefly, the sections were thawed and fixed in 4% paraformaldehyde, rinsed in phosphate buffered saline (PBS) and treated with 0.25% acetic anhydride in triethanolamine, pH 8.0, and then dehydrated and delipidated, before processing for hybridization. The hybridizations for IL-1β and IL-1ra mRNA were carried out in parallel on sections from all animal treatment groups and time points in each experiment (see Tissue preparation and processing). A cRNA probe for IL-1ra was generated from rat IL-1ra cDNA corresponding to bases 476–881 of rat pro-IL-1ra mRNA. A cRNA probe for IL-1β was generated from rat IL-1β cDNA corresponding to bases 380–930 of rat pro-IL-1β mRNA. Transcription of antisense and sense riboprobes was carried out according to the manufacturer's instructions (Riboprobe System Promega, Promega, Madison, USA) using SP6 and T7 polymerases (Promega) in the presence of ^35^S-uridine triphosphate (specific activity 1000–1500 Ci/mmol, Amersham, Buckinghamshire, UK). The specific activity of the labeled probes was approximately 3.37×10^5^ dpm/ng after the transcription reaction and the purification steps.

The slides were hybridized in a humidified chamber at 50°C for 18 h with 12×10^6^ cpm of the probe/ml hybridization solution. The hybridization mixture contained 50% formamide (v/v), 4×SSC (1×SSC = 0.15 M NaCl/0.015 M sodium citrate), 500 µg/ml single-stranded herring sperm DNA (D-7290, Sigma), 250 µg/ml yeast tRNA (R-5636, Sigma), 1×Denhardt's reagent (0.02% (w/v) ficoll, 0.02% (w/v) polyvinylpyrrolidone, 0.02% (w/v) bovine serum albumin), 10% (w/v) dextran sulphate, 0.02 M Na_2_HPO_4_/NaH_2_PO_4_·xH_2_O (pH 7.0) and 120 mM dithiothreitol. After hybridization, the sections were washed for 60 min in 1×SSC/50% formamide at 50°C followed by a 30 min wash in 1×SSC containing RNase A (20 µg/ml) at 37°C and a final wash in 1×SSC at 50°C for 30 min. The sections were rinsed rapidly in distilled water and 70% ethanol and then dried and exposed to X-ray film (Biomax MR, Eastman Kodak, Rochester, USA) for 4 weeks, or dipped in nuclear track emulsion (K5; Ilford Knutsford Cheshire, UK) and kept at 4°C for 12 weeks. The emulsion was developed in D-19 (Eastman Kodak). Counterstaining with cresyl violet following the hybridization was performed in order to identify the regional distribution of IL-1β and IL-1ra mRNA expression.

No hybridization signal was found in brain- and spinal cord sections hybridized with sense cRNA probes, or after pretreatment of tissue sections with RNAse, or when the IL-1β or IL-1ra cRNA probe was omitted.

### IL-1β and IL-1ra immunohistochemistry

Perfusion-fixed, cryostat sections were thawed, rinsed in 0.1 M Tris-buffered saline (TBS, pH 7.6), incubated with TBS containing 0.3% H_2_O_2_ for 20 min and then rinsed in TBS containing 0.5% Triton X-100 (TBS-T). Thereafter, the sections were preincubated for 1 h with TBS-T containing 5% BSA and 2% normal goat serum for the detection of IL-1β, or 5% BSA and 2% normal donkey serum for the detection of IL-1ra. The sections were incubated for 48 h at 4°C in preincubation buffer containing a monoclonal mouse anti rat IL-1β antibody (Silk 5, locally produced, [51]; diluted 1∶200), or a sheep anti rat IL-1ra antiserum (code S373/B1) kindly provided by dr S. Poole (Potters Bar, U.K.;[52]; diluted 1∶50). Thereafter, sections were washed in TBS-T and incubated for 1 h at room temperature with biotinylated goat anti-mouse IgG (1∶400; Jackson, Westgrove, USA) or biotinylated donkey anti-sheep IgG (1∶400; (Jackson) diluted in preincubation buffer. Following washing in TBS-T, the sections were incubated for 1 h at room temperature with avidin-biotin labeled peroxidase (1∶400; Vectastain Elite ABC kit, PK-6101; Vector Laboratories, Burlingame, USA) diluted in preincubation buffer. Peroxidase activity was detected, using 3,3′-diaminobenzidine (DAB, D-5637, Sigma) as a substrate. The sections were dehydrated in graded series of ethanol and subsequently embedded in Entellan mounting medium (Merck, Darmstadt, Germany) and examined under a light microscope (Olympus, Tokyo, Japan).

Omittance of the primary antibodies served as a negative control. The IL-1β immunostaining observed in the present material was confirmed with a polyclonal antibody generated in rabbit to recombinant rat IL-1β (Glaxo, IMB, Geneva Switzerland; [26]) (data not shown). The specificity, sensitivity and absence of cross-reactivity of the IL-1β and IL-1ra antibodies has been demonstrated earlier [30], [52].

### CD68, PLP and CD3 immunohistochemistry

Activated mononuclear cells, demyelination and T-lymphocytes were identified by immunohistochemistry using mouse monoclonal antibodies directed towards CD68 (1∶100, ED1; Serotec, Düsseldorf, Germany; [53]), and to PLP (1∶1000, MCA839G, Serotec, Düsseldorf, Germany) or a rabbit polyclonal antibody directed towards CD3 (1∶50; DAKO, Glostrup, Denmark), respectively in preincubation buffer containing TBS-T, 5% BSA and 2% normal donkey serum or 5% skimmed milk. The immunohistochemical procedure was carried out as described for IL-1β and IL-1ra.

### Histological stainings

In order to visualize gross histology of the tissue, sections were stained with cresyl violet and hematoxylin-eosin. To study myelin degradation products, Oil-Red O neutral lipid staining was carried out on perfusion-fixed sections [39]. Tissue sections were taken to RT, incubated for 10 min in 0.3% Oil-Red O and 60% isopropanol in water and rinsed in tap water. Thereafter, sections were counterstained with Mayer's hematoxylin for 40 sec, and embedded in aqua mounting medium.

### Semi-quantitative RT-PCR of IL-1β and IL-1ra mRNA

To determine the temporal expression of IL-1β and IL-1ra at a more quantitative level, we performed q-PCR analysis on brain stem and cervical spinal cord tissue collected from cr-EAE animals in the first phase of disease (n = 4), and in the relapse (n = 4). Total RNA was extracted from 30 µg of tissue homogenized in 175 µl of SV RNA lysis buffer according to the manufacturer's protocol (SV Total RNA Isolation System, Promega Corporation, USA). Samples of 0.5 µg total RNA were reversely transcribed using Avian Myeloblastosis Virus (AMV) Reverse Transcriptase (Reverse Transcription System, Promega). The absence of contaminating genomic DNA and the efficiency of the RT reactions were checked by q-PCR on RNA and cDNA using GAPDH primers.

Primers were designed using Primer Express Software (PE Applied Biosystem). Details of primer sequences are listed below:

IL-1β forward primer: 5′-AAAGAAGAAGATGGAAAAGCGGTT-3′,

IL-1β reverse primer: 5′- GGGAACTGTGCAGACTCAAACTC-3′,

IL-1ra forward primer: 5′- GAGACAGGCCCTACCACCAG -3′, and

IL-1ra reverse primer: 5′- CGGGATGATCAGCCTCTAGTGT-3′.

GAPDH forward primer: 5′- GAACATCATCCCTGCATCCA-3′.

GAPDH reverse primer: 5′- GCCAGTGAGCTTCCCGTTCA-3′.

Relative mRNA levels were established by q-PCR (SYBR Green PCR, ABI 7700, PE Applied Biosystems, California, USA) as described elsewhere [54]. RT-PCR was carried out in a final volume of 20 µl containing 6.5 ng of cDNA, 1× SYBR Green buffer, 3 mM MgCl2, 0.2 mM dATP, dCTP and dGTP, 0.4 mM dUTP, 0.19 µM of each primer, 0.3 U of AmpliTaq Gold DNA and 0.12 U of Amperase uracil-N-glycosylase (all reagents from PE Applied Biosystems). PCR conditions were: 50°C for 2 min, 95°C for 10 min followed by 40 cycles of 95°C for 15 sec and 60°C for 1 min. Threshold cycle (Ct) values provided an index of the mRNA level. The level of GAPDH mRNA was used as an internal standard to control amplification variations due to differences in starting mRNA concentrations. The relative levels of IL-1β and IL-1ra mRNA for each tissue were calculated from the Ct values obtained for the gene of interest (IL-1β, IL-1ra) and the Ct value for GAPDH using the following formula: Relative mRNA expression of the gene of interest (in % compared to GAPDH) = 2^−(Ct gene of interest-Ct GAPDH)^.

### Statistical analysis

Statistical analyses were performed using the SPSS Version 20 (IBM SPSS, Chicago, IL, USA). Comparisons between 2 groups were performed on normally distributed datasets using unpaired Student's t-test. Values of *p<0.05 were considered statistically significant.

## Results

### Neurological deficits

**Figure 1:**
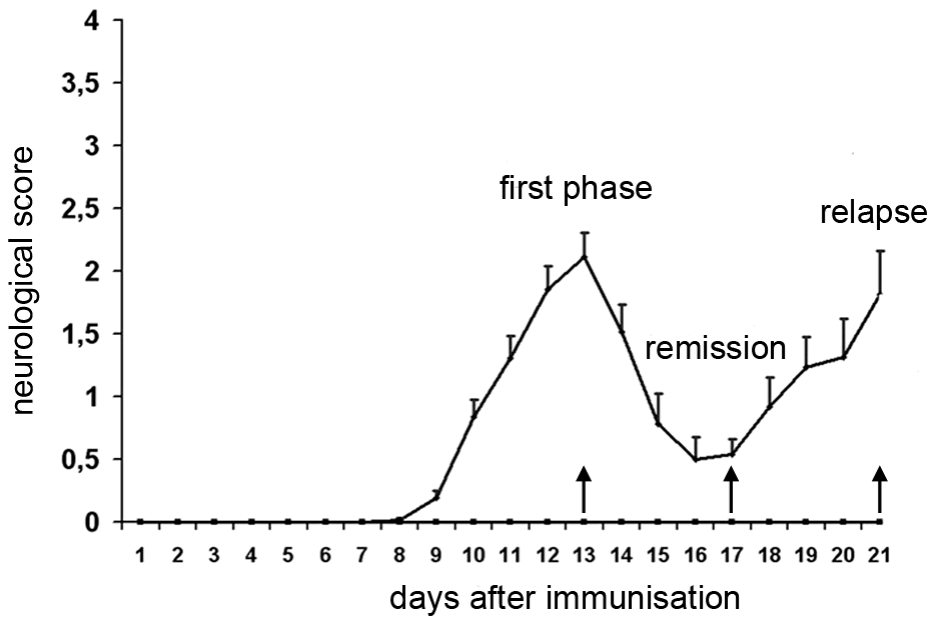
Neurological symptoms during cr-EAE. Male dark agouti rats were immunized with MOG and neurological symptoms were monitored daily. Data represent mean and S.E.M. of neurological scores. Note the typical multiphasic course of the disease with a first phase followed by complete or partial remission and subsequent relapse.

All MOG-treated animals, but none of the controls, developed neurological deficits. As illustrated in figure 1, the mean neurological scores of all cr-EAE animals followed a characteristic multiphasic pattern with a peak of the first disease phase at day 13 (maximal scores 2 or 3 were found in individual animals between day 12 and 15) and a maximal remission of the symptoms at day 17 (scores 0 or 0.5 were seen in individual animals between day 14 and 18). The relapse with maximal scores between 1 and 3 was found in individual animals between day 19 and 21. At that time-point we were obliged to sacrifice all remaining cr-EAE animals as agreed with the local animal ethical committee.

### Histopathological features of CNS lesions

**Figure 2:**
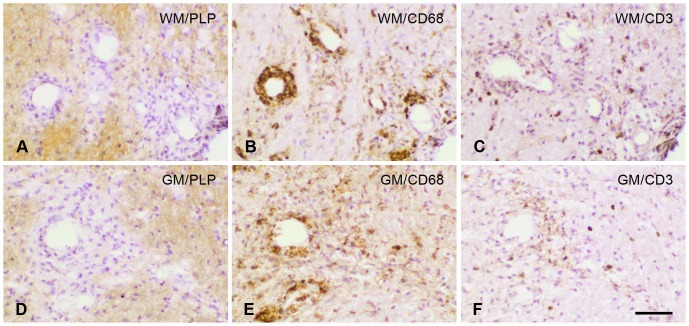
Histopathological features of CNS lesions during the first disease phase of cr-EAE. Left panels: PLP immunoreactivity. Middle panels: CD68 immunoreactivity. Right panels: CD3 immunoreactivity during the first disease phase of cr-EAE in (**A–C**) the trigeminal tract (WM), and (**D–F**) the trigeminal nucleus (GM). Scale bar = 60 µm.

PLP and CD68 stainings were used as markers of demyelination and actived mononuclear cells, respectively. Demyelination was sparse at the early stages of disease studied, which is consistent with previous observations in this animal model [46], [55]. Some periventricular and perivascular loss of PLP staining could be detected in WM and GM areas (Fig. 2A, D), together with a prominent precence of CD68 immunopositive cells (Fig. 2B, E), which is indicative of an early starting demyelinating process. CD3 positive T-lymphocytes were present in the brain and spinal cord of cr-EAE animals (Fig. 2C, F), at similar positions as CD68 immunopositive microglia and/or macrophages. However, the CD3 positive cells were clearly outnumbered by CD68 positive cells present at the sites of inflammation.

**Figure 3:**
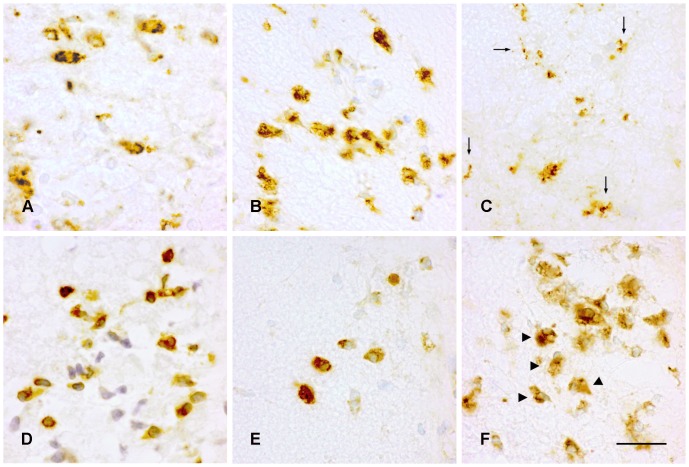
CD68 positive cells in the brain stem and the spinal cord during cr-EAE. Left panels: first phase of disease, middle panels: remission, and right panels: relapse. **(A–C)** CD68 positive cells in the brain stem, and **(D–F)** in the spinal cord. Note the difference in the morphology of CD68 positive cells in the brain stem and spinal cord during the relapse phase. Scale bar = 20 µm. Arrows in **C** indicate ramified CD68 positive cells; arrowheads in **F** indicated cells with an amoeboid morphology.

Unlike PLP and CD3 positive cells, a time- and CNS region-dependent difference in morphology of CD68 positive cells was observed. Within the brain the CD68 positive cells turned into a more ramified microglial-like morphology during the relapse phase (Fig. 3A–C), whereas in the spinal cord, the CD68 positive cell population remained to have an amoeboid-like morphology throughout the different phases of cr-EAE (Fig. 3D–F).

### IL-1β and IL-1ra mRNA expression in brain and spinal cord

**Figure 4:**
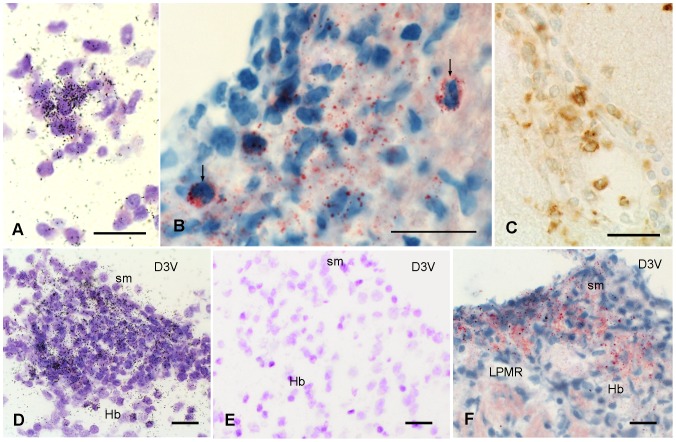
IL-1β expression, CD68 immunoreactivity and Oil-Red O staining. Expression of mRNA for IL-1β, presence of activated macrophages/microglial cells, and lipid fragmentation. All pictures were taken from animals at the first phase of cr-EAE. **(A)** IL-1β mRNA in cerebellar lobule. **(B)** Oil-Red O in cerebellar lobule. Arrows indicate lipid laden cells. **(C)** CD68 in cerebellar lobule. **(D)** IL-1β mRNA in the habenula. **(E)** Absence of IL-1β mRNA in the habenula of a control animal **(F)** Oil-Red O in the habenula. Sections are counterstained with Cresyl Violet **(A, D)** or with Mayer's hematoxylin **(B, E, F)** D3V: dorsal third ventricle, Hb habenula nuclei, LPMR: lateral posterior thalamic nucleus, sm: stria medullaris. Scale bars = 20 µm.

*In situ* hybridization revealed expression of IL-1β and IL-1ra mRNA in white matter structures as well as in specific grey matter areas (Fig. 4A, D) in the brain and spinal cord of cr-EAE, also showing positive CD68 (Fig. 4C) and Oil-Red O staining (Fig. 4B, F). Control animals did not show IL-1β and IL-1ra mRNA (Fig. 4E), nor did normal appearing WM or GM, i.e. in the absence of CD68 positive cells or demyelination.

#### Regional distribution

**Figure 5:**
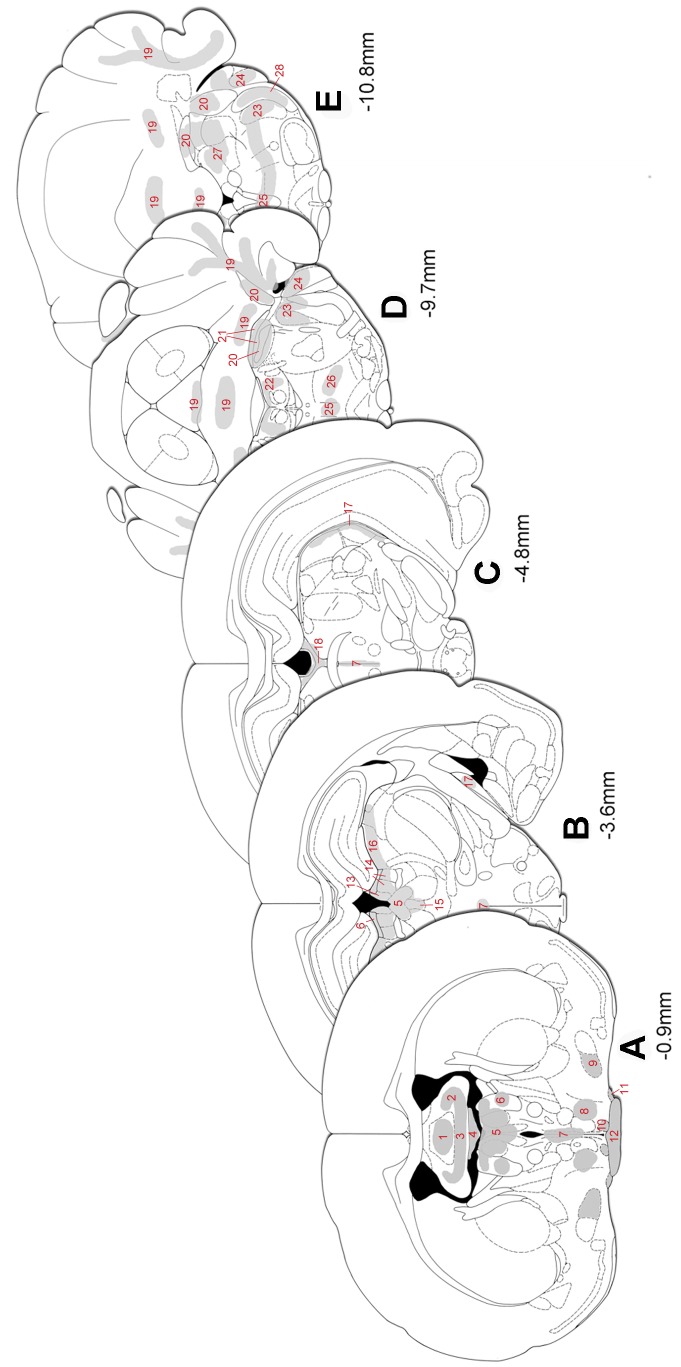
Distribution of IL-1β and IL-1ra mRNA in affected brain regions of cr-EAE rats. Grey areas represent localization of IL-1β and IL-1ra mRNA in the **(1)** triangular septal nucleus, **(2)** septofimbrial nucleus, **(3)** ventral hippocampal commissure, **(4)** subfornical organ, **(5)** paraventricular thalamic nucleus, **(6)** stria medullaris of the thalamus, **(7)** 3^rd^ ventricle, **(8)** medial preoptic area, **(9)** nucleus of diagonal band, **(10)** suprachiasmatic nucleus, **(11)** supra optic hypothalamic nucleus, **(12)** optic chiasm, **(13)** medial habenular nucleus, **(14)** lateral habenular nucleus, **(15)** intermediodorsal thalamic nucleus, **(16)** lateral thalamic nuclei, **(17)** optic tract, **(18)** habenular commissure, **(19)** cerebellar lobules, **(20)** cerebellar peduncles, **(21)** parabrachial nucleus, **(22)** central grey pons, **(23)** trigeminal nucleus, **(24)** cochlear nucleus, **(25)** predorsal bundle, **(26)** pontine reticular nucleus, **(27)** vestibular nucleus, and **(28)** spinal trigeminal tract.

An overview of the regional distribution of IL-1β and IL-1ra mRNA expressing cells is given in figure 5 and table S1.

**Figure 6:**
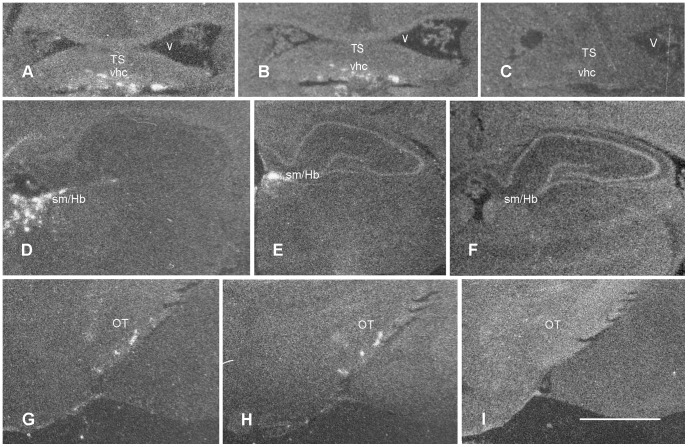
IL-1β and IL-1ra mRNA in first phase and remission of cr-EAE. Film autoradiographs of affected brain regions in cr-EAE rats after *in situ* hybridization for IL-1β (left pannels) or IL-1ra (middle pannels) or with sense probes (right panels). Upper and middle pannels are taken from animals in first disease phase, lower panels from animals in remission. **(A, B)** clusters of IL-1β and IL-1ra mRNA in triangular septal nucleus (TS) and ventral hippocampal commissure (vhc); **(D, E)** clusters of IL-1β and IL-1ra mRNA in stria medullaris of the thalamus (sm) and habenular nuclei (Hb), and **(G, H)** IL-1β and IL-1ra mRNA in optic tract (OT). **(C, F)** sections hybridized with a sense probe for IL-1β, (**I**) section hybridized with a sense probe for IL-1ra. Scale bar (**A–I)** = 2 mm.

**Figure 7:**
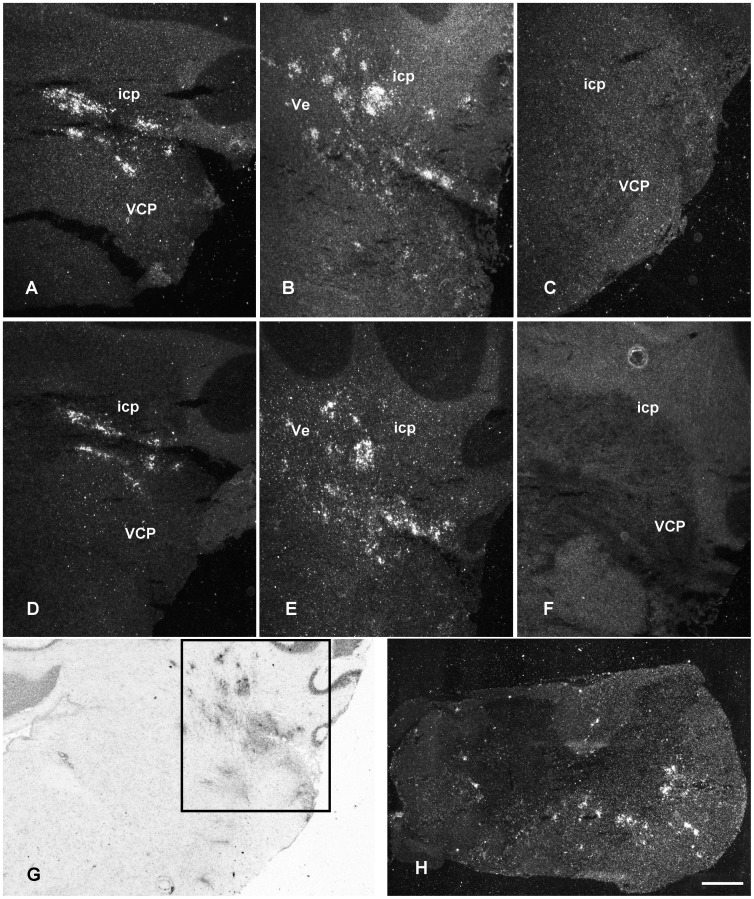
IL-1β and IL-1ra mRNA in brain stem and spinal cord during cr-EAE. Upper pannels IL-1β mRNA. Middle pannels: IL-1ra mRNA. **(A, D)** First phase of disease; **(B, E)** remission; **(C, F, H**) relapse. **(G)** anatomical localization of CD68 positive cells during the first disease phase in the lateral brain stem areas including inferior cerebellar peduncles, interpeduncular nuclei, cochlear nuclei, lateral vestibular nucleus and trigeminus. Note the absence of IL-1β and IL-1ra mRNA in the brain stem during the relapse (**C, F**), while IL-1β and IL-1ra signal is still present in the spinal cord during the relapse **(H)**. Frame in G refers to brain stem areas shown in A–F. Scale bar **(A–H**) = 500 µm. Icp = inferior cerebellar peduncle; VCP  =  ventral chochlear nucleus, posterior; Ve  =  vestibular nucleus.

Analysis of the autoradiographs and emulsion film exposed brain sections revealed IL-1β and IL-1ra mRNA in affected WM areas, e.g. the ventral hippocampal commissure (Fig. 6A, B), the stria medullaris thalamus and the optic chiasma, in the forebrain (level −0.9 mm; Fig. 5A), and in the midbrain (bregma levels −3.6, −4.8 mm; Fig. 5B, C), e.g. the optic tract (Fig. 6G, H). In addition, in the fore- and midbrain affected GM areas showed IL-1β and IL-1ra mRNA expressing cells in septal nuclei (Fig. 6A, B), habenular nuclei (Fig. 6D, E), nuclei of the thalamus, and in hypothalamic nuclei including, suprachiasmatic, supraoptic and medial preoptic nuclei. As a control, hybridization with the sense probe did not result in labeled cells (Fig. 6C, F, I). In the brain stem (bregma levels −9.7, −10.8 mm; Fig. 5D, E), at sites with prominent CD68 immunoreactivity (Fig. 7G), IL-1β and IL-1ra expressing cells were present along fiber tracts (WM) and associated nuclei (GM), *i.e.* the trigeminal areas, cochlear nuclei, vestibular nuclei, parabrachial nuclei, the predorsal bundle and the pontine reticular nucleus (Fig. 7A, B, D, E). In addition, IL-1β and IL-1ra expressing cells were found in affected cerebellar white matter, in cerebellar peduncles and in cerebellar lobules 1–3 (Fig. 7A, B, D, E). Note the absence of IL-1β and IL-1ra expression during the relapse of disease (Fig. 7C, F). Emulsion film exposed sections and cresyl violet counterstaining further revealed isolated labeled cells in ventricular choroid plexus and in meninges. Within the spinal cord, autoradiographs and emulsion film exposed sections and cresyl violet counterstaining showed that IL-1β and IL-1ra expressing cells occur in affected white matter areas at the cervical, thoracal, lumbar and sacral level of the spinal cord, primarily in the dorsal, ventral and lateral funiculi, often associated with bloodvessels (Fig. 7H).

As indicated in table S1, the relative quantity of IL-1β and IL-1ra mRNA labeled cells was most pronounced in the stria medullaris thalamus of the diencephalon and in the brain stem pons regions, less pronounced in the forebrain septal regions, the ventricular choroid plexus, cerebellar white matter and in the spinal cord white matter, and the least present in the optic areas, subfornical organ, thalamus and hypothalamus. Based on the relative quantities determined, the amount of IL-1β and IL-ra expressing cells did not differ clearly from eachother within a brain region studied.

#### Cellular distribution

**Figure 8:**
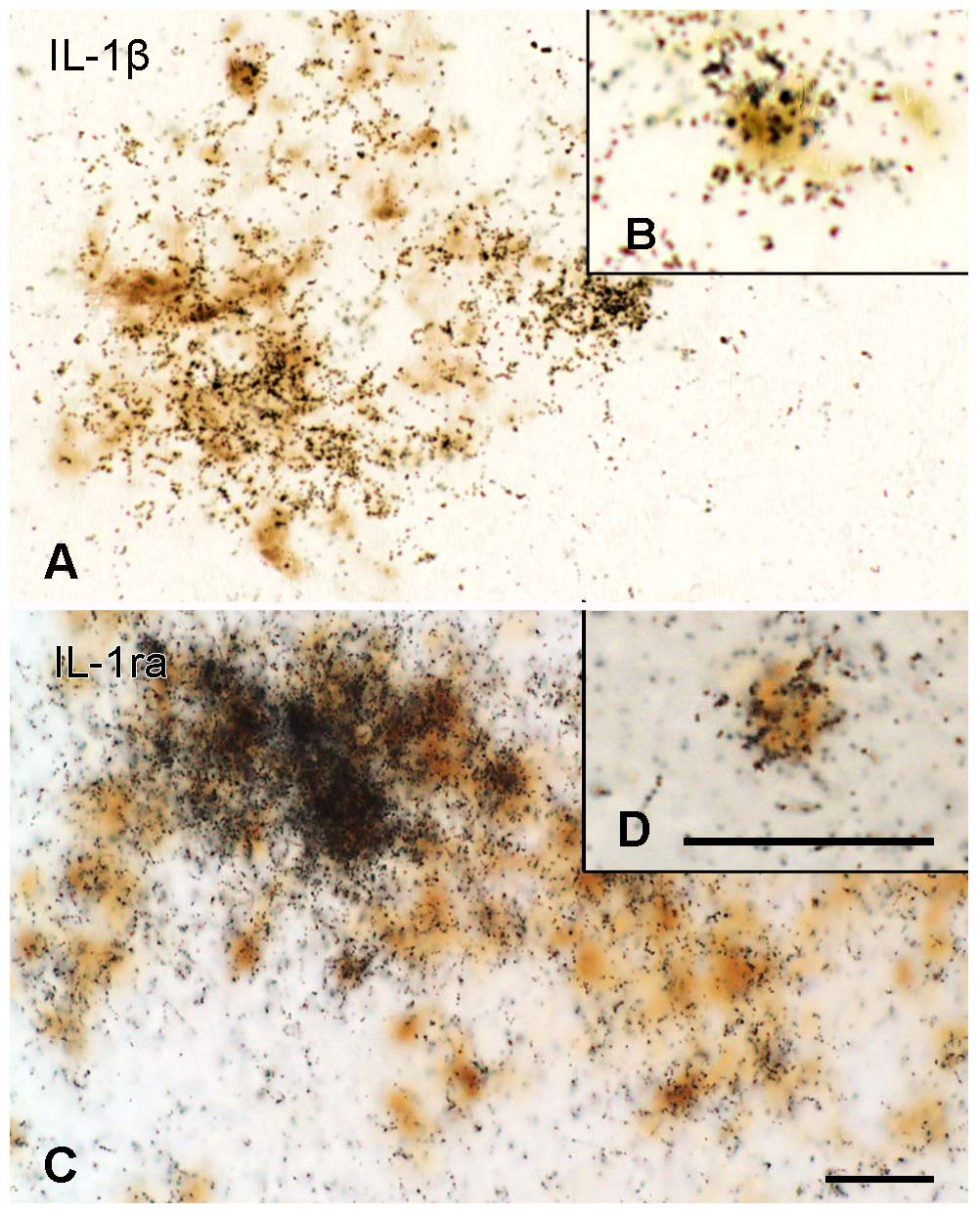
IL-1β and IL-1ra mRNA in CD68 positive cells. Expression of IL-1β and IL-1ra mRNA in CD68 positive activated macrophages/microglial cells during the first disease phase of EAE. **(A, B)** IL-1β mRNA (black grains) combined with CD68 immunoreactivity (brown) in the vestibular nucleus of the brain stem. **(C, D**) IL-1ra mRNA (black grains) combined with CD68 immunoreactivity in the spinal cord. Scale bars = 20 µm.

Double labeling studies revealed co-localization of IL-1β (Fig. 8A, B) or IL-1ra mRNA (Fig. 8C, D) with CD68, demonstrating that macrophages and/or endogenous activated microglial cells expressed either or both of these cytokines. Moreover, in sections processed for *in situ* hybridization, all IL-1β and IL-1ra mRNA expressing cells were CD68 positive and were often associated with cells having a round or oval nucleus, that were smaller and more intensely stained than other IL-β/IL-ra mRNA negative cells.

#### Temporal distribution

In the brain and spinal cord, analysis of the autoradiograms and emulsion exposed sections revealed the presence of IL-1β and IL-1ra mRNA during the first phase of the disease, and in the remission phase (Table S1; Fig. 7A, B, D, E). In contrast, during the relapse far less signal was found at any of the brain levels studied (Table S1; Fig. 7C, F), whereas IL-1β and IL-1ra mRNA remained present in the spinal cord at this stage (Table S1; Fig. 7H).

### Semi-quantitative RT-PCR of IL-1β and IL-1ra mRNA

**Figure 9:**
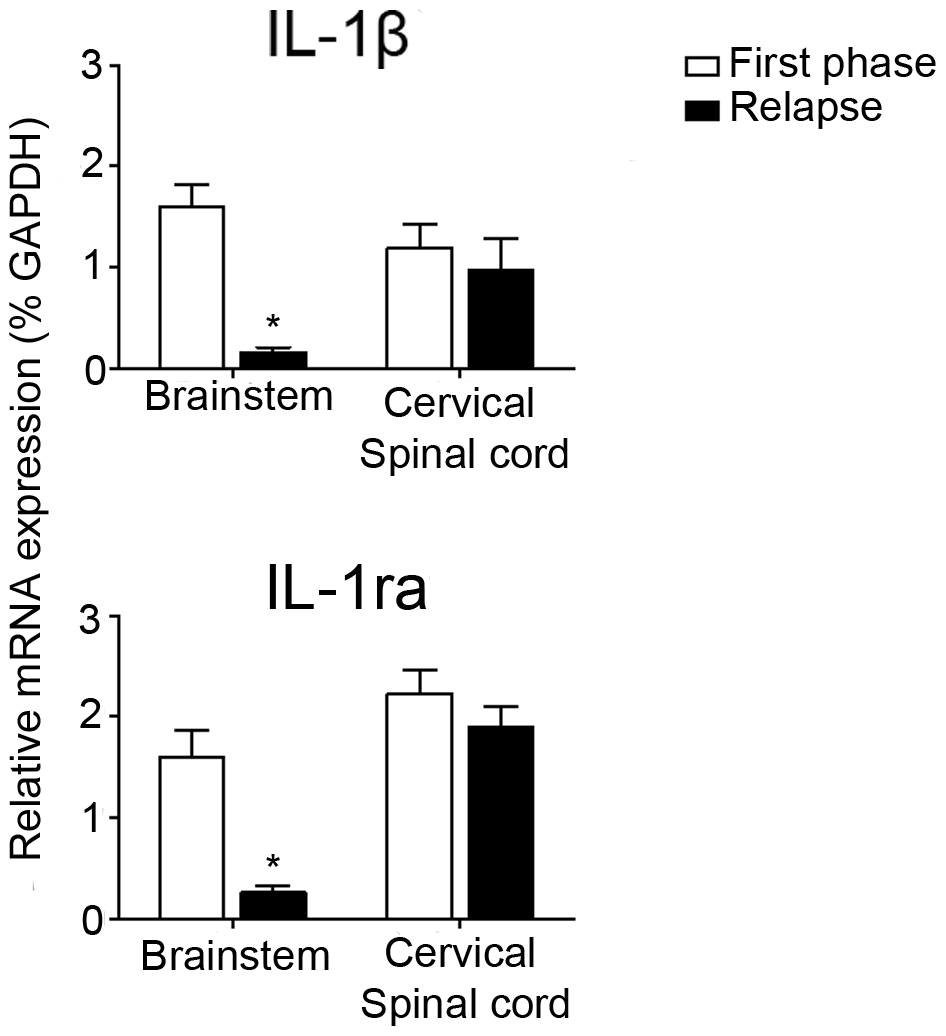
Semi-quantitative RT-PCR analysis of IL-1β and IL-1ra mRNA. Q-PCR of IL-1β and IL-1ra mRNA in brainstem and spinal cord during the first phase and relapse of cr-EAE. Data represent mean ± S.E.M. (n = 4) and are expressed relative to GAPDH mRNA. *P<0.05 vs first phase.

To substantiate the observation that brain and spinal cord differ in temporal expression of IL-1β and IL-1ra in a more quantitative manner, a qPCR experiment was performed. Brain stem and cervical spinal cord clearly express IL-1β and IL-ra during the first phase of disease. However, during the relapse, the levels of IL-1β and IL-ra mRNA were significantly reduced in the brain stem, whereas in the cervical spinal cord they were still present to the same extent as in the first phase of disease (Fig. 9).

### Immunohistochemistry of IL-1β and IL-1ra in brain and spinal cord

**Figure 10:**
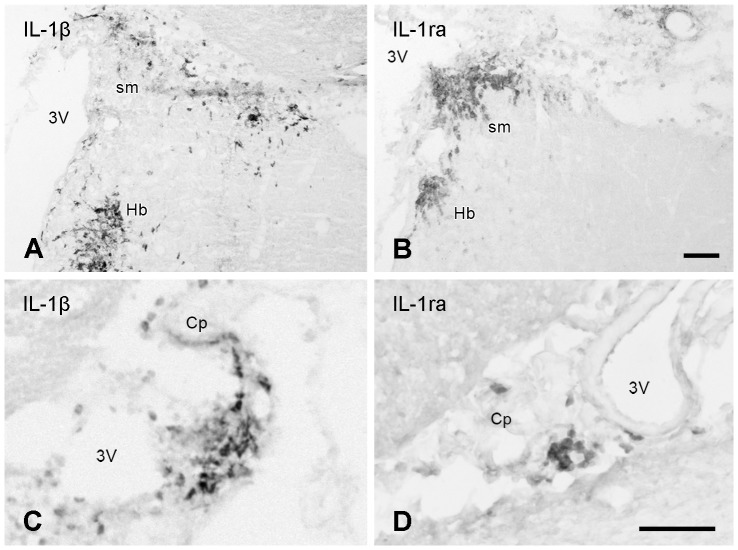
IL-1β and IL-1ra immunoreactivity during the first disease phase of cr-EAE. In the midbrain, clusters of IL-1β positive cells **(A, C)** and IL-1ra positive cells **(B, D)** were found in **(A, B)** affected areas close to the 3rd ventricle (3V) including the stria medullaris thalami (sm) and the medial habenula (Hb), and **(C, D)** in the choroid plexus Scale bar **(A, B)** = 50 µm. Scale bar (**C, D**) = 30 µm.

IL-1β and IL-1ra immunohistochemistry was performed to determine whether the appearance of IL-1β and IL-1ra mRNA during early cr-EAE translates into protein. Similar to their mRNA, the relative number of IL-1β positive cells did not overtly differ from that of IL-1ra positive cells (Fig. 10A-D). The IL-1β and IL-1ra immunopositive cells occur in CNS areas close to the cerebral ventricles, including the habenula and the stria medullaris (Fig. 10A, B), and in the choroid plexus (Fig. 10C, D).

## Discussion

The present study is the first to demonstrate that during the early clinical phases of experimental MS, i.e. cr-EAE, IL-1β and IL-1ra mRNA and protein are not only expressed in white matter, but also in specific grey matter areas within the CNS, which are also positive for CD68 and Oil-Red O. The IL-1β and IL-1ra mRNA expressing cells were identified as macrophages and/or endogenous activated microglial cells.

In more recent years, it has become evident that within the CNS of MS patients besides white also grey matter lesions are present, which can explain more extensively certain neurological and psychiatric symptoms observed in those patients. As inflammatory processes take part in the pathogenesis of MS, we questioned whether an important inflammatory mediator, IL-1β and, its functional counteracting partner, IL-1ra are present in affected WM and GM regions in the CNS during cr-EAE in DA rats, an experimental animal model, mimicking some pathological aspects of relapsing-remitting MS. Indeed, inflammatory processes, i.e. an influx of monocytes, and to a lesser extent T-cells, as well as activation of local microglial cells are clearly present in WM and GM at the early stages of cr-EAE studied. Moreover, some demyelination is observed, but limited to periventricular and perivascular locations at these time-points. These observations are in accordance with previous studies showing that demyelination is sparse whereas inflammation is prominent during early cr-EAE in DA rats [46], [55]. Although this may be a limitation of the model used, inflammatory mediators, including IL-1β, are known to be upregulated early in inflammatory processes [26]–[29] and contribute to the subsequent process of demyelination [32]–[36]. By using *in situ* hybridization and immunohistochemical approaches, we were able to detect IL-1β and IL-1ra expressing cells in cr-EAE affected GM regions. In addition, cerebral white matter fiber bundles and WM in the spinal cord were affected and showed IL-1β and IL-1ra expressing cells. Within brain regions, most expression was detected close to veins or ventricles. The presence of IL-1β and IL-1ra mRNA was observed in close association, regionally and temporally, with the occurrence of infiltrating monocytes/activated microglia and was absent in control rats or normal appearing WM and GM.

The appearance of IL-β and IL-1ra expressing cells in WM areas within the brain of our experimental MS model is consistent with elevated IL-1β [38], [56] and IL-1ra [39] expression in active WML in post-mortem brain material of MS patients and of the marmoset EAE model for MS [57]. Furthermore, IL-1β and IL-1ra production within the ventricular choroid plexus is in line with observations that IL-1β production within the choroid plexus is significantly increased during the early phase of EAE in mice [58]. Moreover, IL-1β [59], [60] and IL-1ra [59] protein levels are significantly enhanced in the cerebrospinal fluid (CSF) of MS patients. As the choroid plexus regulates the composition of the CSF content, it may be suggested that choroid plexus-derived IL-1β and IL-1ra are secreted into the CSF [61].

The combination of IL-1β or IL-1ra mRNA *in situ* hybridization histochemistry with immunohistochemistry for CD68 indicated that infiltrating macrophages and/or activated endogenous microglial cells in WM and GM are the main sources of IL-1β and IL-1ra mRNA production during conditions of cr-EAE, which is in line with research showing increased expression of IL-1β in activated microglia and macrophages in EAE [41], [62], [63] and in MS [38]. Although we cannot exclude the possibility that IL-1β or IL-1ra is expressed by T-cells or endothelial cells, our data suggest that if so, it will be a minor contribution to the production of IL-1β or IL-1ra during cr-EAE. Though the presence of the IL-1 type I receptor in brain endothelial cells has been considered to be of importance in EAE [64], and in immune-to-brain communication in more general [65].

The present *in situ* hybridization studies showed that the induction of IL-1β and IL-1ra mRNA and protein in the CNS appeared to be persistent but is clearly diminished during the relapse of cr-EAE, whereas IL-1β and IL-1ra expression in the spinal cord remained elevated during the relapse. These results were supported by qPCR analysis on brain stem and spinal cord tissue obtained at different stages of cr-EAE showing statistically significant differences in IL-1β and IL-1ra expression between brain stem and spinal cord during the relapse. This discrepancy in disease phase-related expression between the brain and spinal cord can possibly be explained by a reduced inflammatory phenotype in the brain, and/or a functional and/or morphological difference in microglial subtypes in brain versus spinal cord as suggested previously [66]. As there is rather limited demyelination in the CNS at these relative early stages of cr-EAE, it is unlikely that the observed difference is due to variation in the demyelination process between brain and spinal cord, which we also did not notice. Thus, we consider the appearance of IL-1β and IL-1ra mRNA to be most clearly associated with inflammation during early cr-EAE, but they can also play a role in the starting demyelination process. Indeed, myelin degradation products were found to be present together with CD68 positive cells and IL-1β and IL-1ra protein expressing cells in WM and GM within the affected CNS areas. The lipid-laden macrophages and/or microglial cells and the lipid fragments observed likely represent the early stages of lesion formation, i.e. starting demyelination [67], [68]. Also in biopsy GM tissue of early MS patients, signs of inflammatory activity and ongoing demyelination were observed [20] which is less evident in MS post-mortem GML. Therefore, our observations in the cr-EAE model most likely represent early stages of MS.

A contributing role of IL-1β in demyelination has been supported by the observations that overexpression of IL-1β in mouse striatum led to demyelination in this brain area [69], and treatment of oligodendrocytes with IL-1β *in vitro* resulted in oligodendrocyte damage [70]. In addition to WM damage, IL-1β expression is also considered to be involved in GM damage [24]. This has become evident by *in vitro* [71]–[73], EAE [74] and other *in vivo* animal studies in which IL-1β was injected intracerebrally in rat leading to apoptotic cell death [75] or axonal injury [76], [77]. Thus the observed presence of IL-1β in WM and GM regions within the CNS of our cr-EAE rats may contribute to oligodendrocyte damage, demyelination and subsequent axonal loss and thus could account for several of the neurological symptoms described in EAE, including paralysis [78], [79], enhanced pain sensation [79]–[81] and loss of vision [82]–[85]. Similarly, in MS patients, impaired vision, sensory and motor functions are symptoms that can manifest themselves by lesions within the optic tract [86], [87], tracts within the brain stem [88], [89], cerebellum [90] and spinal cord [91]–[94], respectively [95].

In addition, GM lesions could account for symptoms as depression [96], [97] and impaired cognition [98], [99] in animals suffering from EAE. In comparison, approximately 50% of MS patients experience cognitive deficits [100], [101], including problems with spatial learning and working memory [100]. Although we realize that cognitive processes are mediated by communication of various brain regions and involve multiple neurotransmitter systems, we now focus on an important brain region involved in learning and memory which is the hippocampus [102]. The hippocampus receives cholinergic input from the habenular and septal nuclei [103], [104]. Besides local effects of lesions in the e.g. habenula itself, also its affected projections towards the hippocampus could account for alterations in cholinergic innervation and subsequent impaired memory function [105]. As such, lesions in the habenular and septal nuclei, as observed in our cr-EAE rats, could explain decreased levels of choline acetyltransferase (ChaT) in the hippocampus as seen in EAE [98] and in MS patients [106]. We thus speculate that, besides an indirect effect of IL-1β on neurological functioning by promoting oligodendrocyte and neuronal damage in WM affected areas, IL-1β produced within GM lesions may contribute to neuronal dysfunction by directly influencing neurotransmission locally or at distant projection sites. In support of this speculation, we observed IL-1β in nuclei within the basal forebrain (septal nuclei), within the diencephalon (medial habenula nucleus and thalamic nuclei) and within the brainstem (trigeminal, vestibular, parabrachial and reticular nucleus), i.e. in regions where cholinergic neurons are present [107], [108]. In addition, experimental animal studies show that centrally administered IL-1β reduces acetylcholine (Ach) release from the hippocampus, which coincided with memory impairment [109], [110]. Additionally, in a transgenic mouse model for Alzheimer's disease, beta-amyloid induced upregulation of IL-1β within the CNS coincided with reduced numbers of ChaT positive neurons and attenuated Ach release [111].

Although damaging effects of IL-1β within the CNS have been shown to be counterbalanced by IL-1ra treatment [112]–[114], it is conceivable that the balance in IL-1β and IL-1ra production, rather than the IL-1β levels per se, determines IL-1β signaling and thereby its potential detrimental effects [73], [115]. Therefore, our observation of relatively equal expression of IL-1β and IL-1ra within the CNS may suggest that the levels of IL-1ra produced are too little to overcome the detrimental effect of IL-1β. This is supported by *in vitro* and *in vivo* studies showing that relative high quantities of IL-1ra have to be administered to neutralize an IL-1β induced effect [110], [116]–[119] although the affinity of both ligands for the type I receptor is similar [116], [118]. Thusfar, a clinical trial using IL-1ra has not been performed in MS patients, although it has been shown to be effective in EAE animals [43], [44]. Of interest to note is that regular treatment of MS patients with steroids, IFN-β or glatiramer acetate raised serum levels of IL-1ra [59], [120]–[122] and IFN-β, and also enhances microglial production of IL-1ra [123]. We therefore consider an initial clinical trial to treat MS patients with Anakinra, a recombinant form of IL-1ra, is warranted in MS patients, in which, next to the traditional read-outs, also grey matter pathology and related symptoms should be taken into account.

In summary, the present study showed the appearance of IL-1β and IL-1ra in various specific GM regions, in addition to white matter areas, affected during cr-EAE. The expression is dedicated to CD68 positive macrophages/activated microglial cells in areas with starting demyelination. These observations suggest that IL-1β contributes to the development of GM lesions, in addition to WM lesions, during cr-EAE which cannot be counteracted efficiently by IL-1ra. Moreover, IL-1β localized in specific nuclei can play a role in local or distant neurological impairment by affecting neurotransmitter production and/or action, of e.g. the cholinergic system. Thus, the localization of IL-1β in GM lesions may explain some clinical deficits which cannot be attributed solely to the presence of IL-1β in WM lesions, and is therefore of potential interest for the pathogenesis and treatment of MS patients with GM lesion-related deficits.

## Supporting Information

Table S1 **Semi-quantification of IL-1β and IL-1ra expressing cells in CNS regions during the course of cr-EAE.** The levels of IL-1β and IL-1ra mRNA labeled CD68 positive cells are: no labeled cells (0), >33% of cells is labeled (1), 33–66% of cells is labeled (2), >66% of cells is labeled (3).
(DOC)
