# Supplementary material for: Interleukin-1β and Interleukin-1 Receptor Antagonist Appear in Grey Matter Additionally to White Matter Lesions during Experimental Multiple Sclerosis
Source: PLoS One. 2013 Dec 23;8(12):e83835. doi: 10.1371/journal.pone.0083835 (PMC3871572; doi:10.1371/journal.pone.0083835)
Supplement: Table S1 — Semi-quantification of IL-1β and IL-1ra expressing cells in CNS regions during the course of cr-EAE. The levels of IL-1β and IL-1ra mRNA labeled CD68 positive cells are: no labeled cells (0), >33% of cells is labeled (1), 33–66% of cells is labeled (2), >66% of cells is labeled (3). (DOC) [file pone.0083835.s001.doc]

**Table S1. Semi-quantification of IL-1β and IL-1ra expressing cells in CNS regions during the course of cr-EAE.**

|  | **First phase** | **Remission phase** | **Relapse phase** |
| --- | --- | --- | --- |
| **Septum** |  |  |  |
| nucleus of diagonal band | 2 | 2 | 0 |
| Triangular septal nucleus | 2 | 2 | 0 |
| Septofimbrial nucleus | 2 | 2 | 0 |
| **Cerebral tracts** |  |  |  |
| Stria medullaris thalami | 3 | 3 | 0 |
| Ventral hippocampal commissure | 2 | 2 | 0 |
| Habenular commissure | 2 | 2 | 0 |
| Optic chiasm | 1 | 1 | 0 |
| Optic tract | 1 | 1 | 0 |
| **Habenular nuclei** |  |  |  |
| Medial habenular nucleus | 2 | 2 | 0 |
| Lateral habenular nucleus | 2 | 2 | 0 |
| **Ventricular choroid plexus** | 2 | 2 | 0 |
| **Circumventricular organs** |  |  |  |
| Subfornical organ | 1 | 1 | 0 |
| **Thalamus** |  |  |  |
| Mediodorsal thalamic nucleus | 1 | 1 | 0 |
| Intermediodorsal thalamic nucleus | 1 | 1 | 0 |
| Lateral thalamic nuclei | 1 | 1 | 0 |
| Paraventricular thalamic nucleus | 1 | 1 | 0 |
| **Hypothalamus** |  |  |  |
| Supraoptic nucleus | 1 | 1 | 0 |
| Suprachiasmatic nucleus | 1 | 1 | 0 |
| Medial preoptic area | 1 | 1 | 0 |
| **Brain Stem** |  |  |  |
| Spinal trigeminal tract | 3 | 3 | 0 |
| Trigeminal nucleus | 3 | 3 | 0 |
| Cochlear nucleus | 3 | 3 | 0 |
| Vestibular nucleus | 3 | 3 | 0 |
| Parabrachial nucleus | 3 | 3 | 0 |
| Central gray pons | 2 | 2 | 0 |
| Predorsal bundle | 3 | 3 | 0 |
| Pontine reticular nucleus | 3 | 3 | 0 |
| **Cerebellum** |  |  |  |
| Cerebellar peduncles | 2 | 2 | 0 |
| Cerebellar lobules | 2 | 2 | 0 |
| **Spinal cord** | 2 | 2 | 2 |

The levels of IL-1β and IL-1ra mRNA labeled CD68 positive cells are: no labeled cells (0), > 33% of cells is labeled (1), 33-66% of cells is labeled (2), > 66% of cells is labeled (3).
